# Engineering Chimeric Hypoallergens for Safer and More Effective House Dust Mite Allergy Vaccines

**DOI:** 10.1111/cea.70197

**Published:** 2025-12-21

**Authors:** Eduardo S. da Silva, Antônio M. S. Fernandes, Elisânia F. Silveira, Raphael C. Silva, Leonardo F. Santiago, Luis F. S. Garcés, Sara Huber, Sabrina Wildner, Tafarel A. Souza, Vitor S. Alves, Lorena M. de Souza, Deise S. Vilas‐Bôas, Peter Briza, Peter Lackner, Luis G. C. Pacheco, Neuza M. Alcântara‐Neves, Fatima Ferreira, Carina S. Pinheiro

**Affiliations:** ^1^ Laboratory of Allergology and Acarology (LAA), Institute of Health Sciences Federal University of Bahia Salvador Brazil; ^2^ Post Graduate Program of Immunology Federal University of Bahia Salvador Brazil; ^3^ Faculty of Health Sciences Technical University of Ambato Ambato Ecuador; ^4^ Department of Biosciences and Medical Biology Paris‐Lodron University of Salzburg Salzburg Austria; ^5^ Laboratory of Nanobiotechnology, Institute of Biotechnology Federal University of Uberlandia Uberlândia Brazil; ^6^ Laboratory of Histotechnology, Department of Biomorphology, Institute of Health Sciences Federal University of Bahia Salvador Brazil

## Abstract

- Chimeric hypoallergens of Der p 1 and Blo t 1 (QBD2/QBD4) reduce IgE reactivity and airway inflammation in mice.
- QBD4 promotes Th1/regulatory responses, suppressing mucus and eosinophilia more effectively than QBD2.

To the Editor,

1

House dust mite (HDM) allergy is a prevalent cause of respiratory allergic diseases, with *Dermatophagoides pteronyssinus* (Der p 1) and *Blomia tropicalis* (Blo t 1) as two of the most clinically relevant species, particularly in tropical and subtropical regions [1, 2]. Allergen‐specific immunotherapy (AIT) remains the only disease‐modifying treatment, but its clinical use is often constrained by the risk of IgE‐mediated side effects induced by native allergen extracts [3]. To overcome these limitations, we designed two chimeric hypoallergenic molecules, QBD2 and QBD4, by integrating Der p 1‐derived T‐cell epitopes into the structural backbone of *Blo t 1.0201*, a natural isoform of Blo t 1, aiming to improve the safety and immunomodulatory efficacy of AIT for patients co‐sensitised to both mite species.

QBD2 and QBD4 were engineered based on the amino acid sequence of *Blo t 1.0201* (AAQ24541), into which immunodominant T‐cell epitopes derived from *Der p 1* (AAB60215) were inserted. The QBD4 construct additionally includes a partial pro‐peptide sequence to potentially enhance structural stability and immunological processing. Recombinant QBD2 and QBD4 were expressed in *Escherichia coli* , purified using affinity and size‐exclusion chromatography and verified by SDS‐PAGE.

To assess their structural integrity and degradation profile, circular dichroism (CD) spectroscopy, endolysosomal degradation assays and enzymatic activity tests were performed. Both QBD2 and QBD4 retained secondary structure features at high temperatures, as shown by CD spectra that remained unchanged even at 95°C, and exhibited high resistance to proteolytic cleavage by endolysosomal enzymes. Enzymatic assays confirmed that both chimeras had negligible protease activity compared to native Der p 1, indicating a reduction in allergenic potential through functional inactivation [4].

The allergenic potential of QBD2 and QBD4 was assessed by measuring IgE reactivity using ELISA with sera from HDM‐allergic individuals. Both chimeras showed significantly reduced IgE binding, with less than 20% of the reactivity observed for Der p 1 (Figure 1A). In basophil activation assays, QBD2 and QBD4 induced minimal degranulation compared to Der p 1, confirming their low allergenic activity. Furthermore, the ability of QBD2 and QBD4 to inhibit IgE binding to Der p 1 in competitive ELISA was negligible, suggesting limited cross‐linking capacity [5].

**FIGURE 1:**
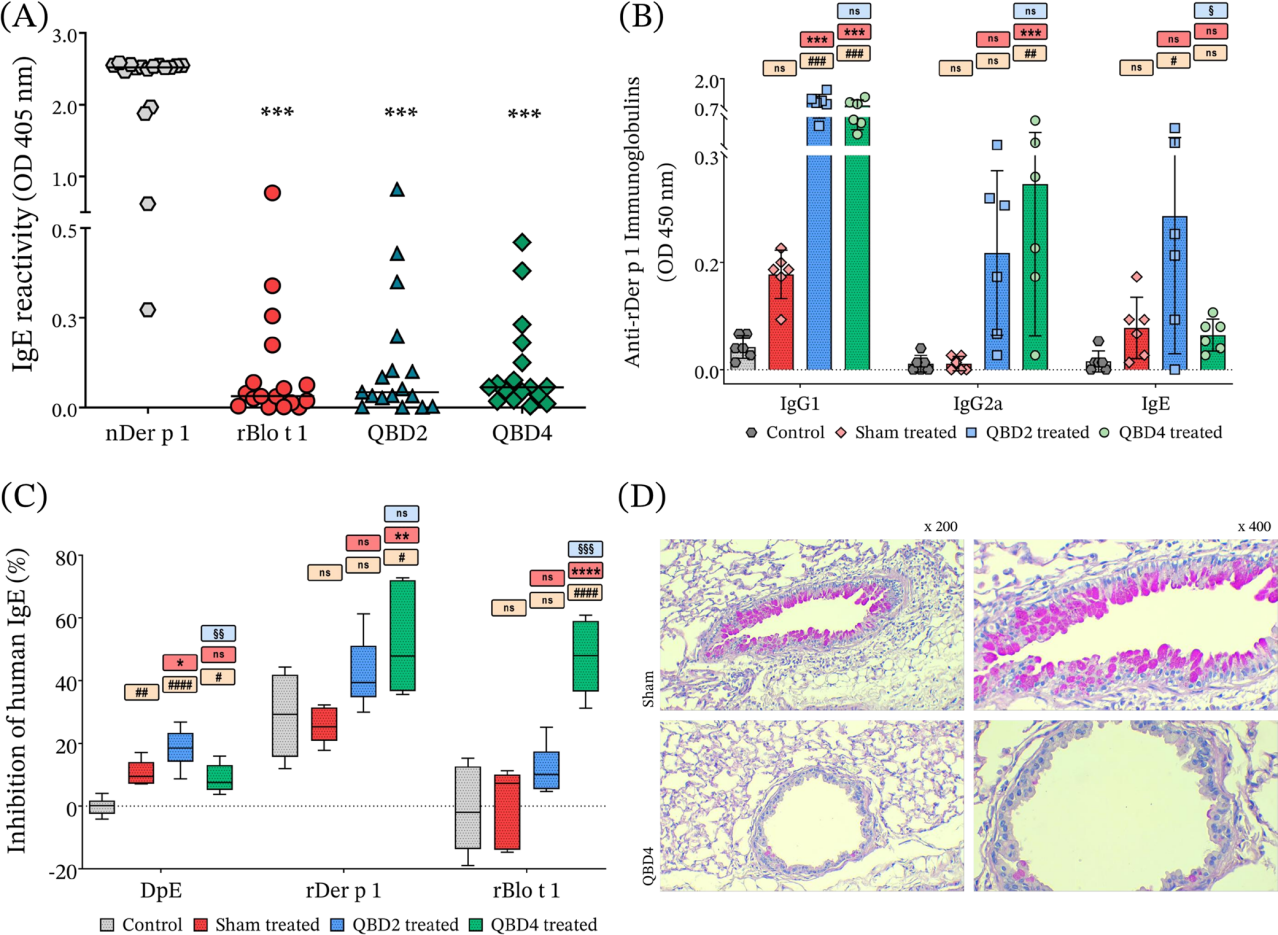
Therapeutic markers of hypoallergenic hybrids designed with segments of group 1 mite allergens. (A) IgE reactivity of hypoallergens in comparison with their parental allergens determined by ELISA (human, *n* = 17). (B) Der p 1 murine‐specific immunoglobulins determined by indirect ELISA. (C) Inhibition of human IgE binding to DpE and parental allergens by sera derived from treated and non‐treated mice. (D) The intracellular mucus and goblet cell hyperplasia staining by the periodic acid–Schiff (PAS) method. One‐way ANOVA with post‐tests of Dunnett or Dunn were used to verify statistical differences. ^#^Comparisons with control, ^#^ *p* < 0.05; ^##^ *p* < 0.01; ^###^ *p* < 0.001; ^####^ *p* < 0.0001. *Comparisons with sham treated, *p < 0.05; **p < 0.01; ***p < 0.001; ****p < 0.0001. ^§^Comparisons with QBD2 treated, ^§^ *p* < 0.05; ^§§^ *p* < 0.01; ^§§§^ *p* < 0.001. DpE, *D. pteronyssinus* extract; Ns, non‐significant; OD, optical density.

To investigate the immunomodulatory potential of these chimeras, an in vivo murine model was employed [4]. A/J mice were sensitised subcutaneously with *D. pteronyssinus* extract (DpE) adsorbed to Alum, followed by intranasal challenge with DpE and subsequently treated subcutaneously with either QBD2 or QBD4. The antibody response revealed that QBD4‐treated mice developed significantly higher levels of Der p 1‐specific IgG2a (a Th1‐associated isotype) and reduced levels of IgE compared to untreated or QBD2‐treated groups (Figure 1B). Importantly, serum from each QBD4‐treated animal was able to block the binding of patients' IgE to Der p 1, indicating the induction of blocking antibodies (Figure 1C).

Analysis of lung cytokine profiles revealed a pronounced shift in the immune response induced by QBD4. Levels of IL‐4 and IL‐13, markers of Th2‐driven allergic inflammation, were significantly reduced, while IFN‐γ (Th1) and IL‐10 (Treg) were upregulated in the lungs of QBD4‐treated mice. In addition, splenocytes from these mice, upon ex vivo restimulation with the hypoallergens, produced increased levels of IFN‐γ and IL‐10, further confirming a skew toward a non‐allergic, regulatory phenotype [6, 7].

Histopathological and cellular assessments supported these findings. Eosinophil and neutrophil counts in bronchoalveolar lavage fluid (BALF) were reduced by more than 50% following QBD4 treatment, accompanied by a marked reduction in leucocyte infiltration, mucus production and goblet cell hyperplasia in airway tissues (Figure 1D). These changes are consistent with reduced airway inflammation and improved tissue homeostasis, reflecting the therapeutic goals of AIT [8].

Overall, QBD4 outperformed QBD2 in inducing a balanced Th1/Treg immune response, aligned with current strategies in hypoallergen design for AIT. Several key features underline its potential as a vaccine candidate [9]. (i) Improved Safety Profile: Both QBD2 and QBD4 demonstrated low IgE reactivity, reducing the likelihood of inducing anaphylactic responses upon administration. (ii) Enhanced Efficacy: The strong induction of blocking antibodies, significant reductions in airway eosinophilia and low mucus production are indicative of clinical efficacy. (iii) Structural Stability: High thermal and endolysosomal stability of QBD4 may contribute to enhanced shelf life and persistent immunogenicity, which are advantageous for vaccine development and distribution.

Despite these promising findings, certain limitations should be acknowledged. The current study did not include a challenge model using *B. tropicalis* ‐derived allergens, which will be addressed in future chronic exposure models to validate long‐term efficacy and specificity. In conclusion, QBD4 represents a promising candidate for next‐generation HDM allergy vaccines. It combines the structural and immunological safety of a hypoallergen with robust immunomodulatory capacity. Future work will focus on dose optimisation, chronic model validation and long‐term assessment of immune tolerance and protection.

## Author Contributions

C.S.P. conceived and supervised the study, performed the experimental work in Austria, coordinated the project in Brazil, secured funding and contributed to writing and revising the manuscript. E.S.S. performed all experiments in Brazil, contributed to data curation, and assisted in writing and revising the manuscript. A.M.S.F., R.C.S., E.F.S., L.F.S., V.S.A., D.S.V.‐B., L.F.S.G., L.M.S., S.H., S.W. and T.A.S. contributed to investigation, experimental procedures and manuscript review. P.B. and P.L. provided laboratory resources and technical support. F.F. offered international supervision, resources and critical revision of the manuscript. N.M.A.‐N. and L.G.C.P. contributed to methodological supervision and manuscript drafting. All authors approved the final manuscript.

## Conflicts of Interest

The authors declare no conflicts of interest.

## Data Availability

All relevant data are available on the ZENODO platform [DOI: 10.5281/zenodo.15839143], or can be obtained from the corresponding author upon reasonable request.
